# Supplementary material for: Combining the Potent Reducing Properties of Pecan Nutshell with a Solvent-Free Mechanochemical Approach for Synthesizing High Ag0 Content-Silver Nanoparticles: An Eco-Friendly Route to an Efficient Multifunctional Photocatalytic, Antibacterial, and Antioxidant Material
Source: Nanomaterials (Basel). 2023 Feb 23;13(5):821. doi: 10.3390/nano13050821 (PMC10005451; doi:10.3390/nano13050821)
Supplement: Supplementary file 1 [file nanomaterials-13-00821-s001.zip › nanomaterials-2223434-supplementary.pdf]

Supporting Information

Combining the Potent Reducing Properties of Pecan Nutshell with a Solvent-Free Mechanochemical Approach for Synthesizing High Ag^0 Content-Silver Nanoparticles: An Eco-Friendly Route to an Efficient Multifunctional Photocatalytic, Antibacterial, and Antioxidant Material

Rita Argenziano †, Sarai Agustin-Salazar †, Andrea Panaro, Anna Calarco, Anna Di Salle, Paolo Aprea, Pierfrancesco Cerruti*, Lucia Panzella* and Alessandra Napolitano

* Correspondence: cerruti@ipcb.cnr.it (P.C.); panzella@unina.it (L.P.)
† These authors contributed equally to this work.

Table of contents

**Figure S1.** (a) XRD patterns of the reaction products synthesized by solvent-assisted bioreductive reaction of $AgNO_3$ using different agrifood byproducts: PNS (red line), grape pomace (blue line), and pomegranate peels and seeds (green line), alongside the amount of Ag^0 and AgCl detected; (b) XRD pattern of the product obtained by mechanochemical treatment of PNS/$AgNO_3$ (85:15 w/w) using vibrational ball milling operating at 60 Hz for 90 min, compared with Ag^0 XRD pattern, demonstrating the presence of AgCl-free Ag^0.	Pag. 2
**Figure S2.** XRD patterns of 85:15 (blue line), 70:30 (green line), and 55:45 (red line) w/w PNS/$AgNO_3$ samples subjected to vibrational ball milling operating at 60 Hz for 90 min.	Pag. 2
**Figure S3.** (a) XRD spectra of PNS/$AgNO_3$ samples subjected to varying planetary ball-milling times (increasing from bottom to top). (b) Dependence of the FWHM of the main peak on the milling treatment time.	Pag. 3

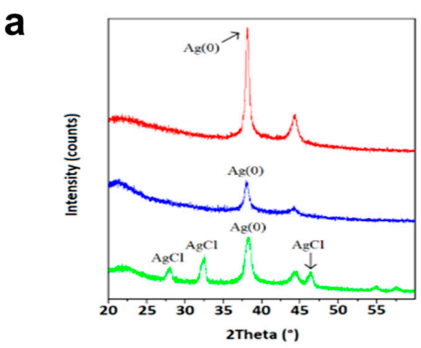

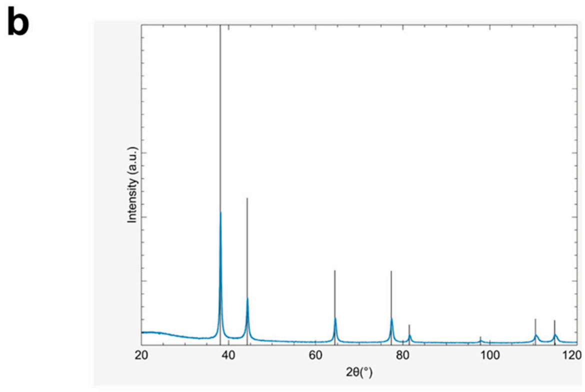

BIOMASS	%Ag(0)	%AgCl
PNS	3.7	0
Grape pomace	2.2	0
pomegranate peels and seeds	6.9	3

Figure S1. (a) XRD patterns of the reaction products synthesized by solvent-assisted bioreductive reaction of $AgNO_3$ using different agrifood byproducts: PNS (red line), grape pomace (blue line), and pomegranate peels and seeds (green line), alongside the amount of Ag^0 and AgCl detected; (b) XRD pattern of the product obtained by mechanochemical treatment of PNS/$AgNO_3$ (85:15 w/w) using vibrational ball milling operating at 60 Hz for 90 min, compared with Ag^0 XRD pattern, demonstrating the presence of AgCl-free Ag^0.

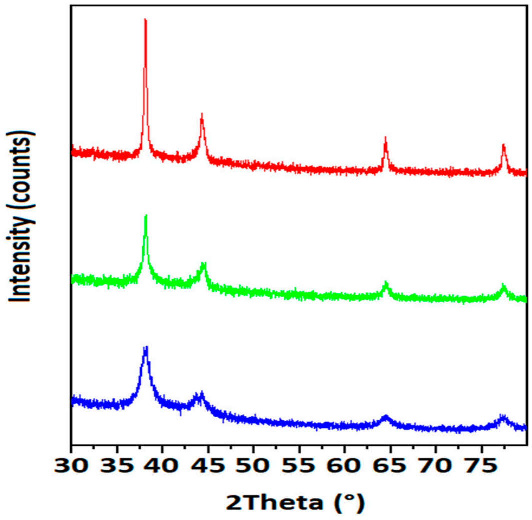

Figure S2. XRD patterns of 85:15 (blue line), 70:30 (green line), and 55:45 (red line) w/w PNS/$AgNO_3$ samples subjected to vibrational ball milling operating at 60 Hz for 90 min.

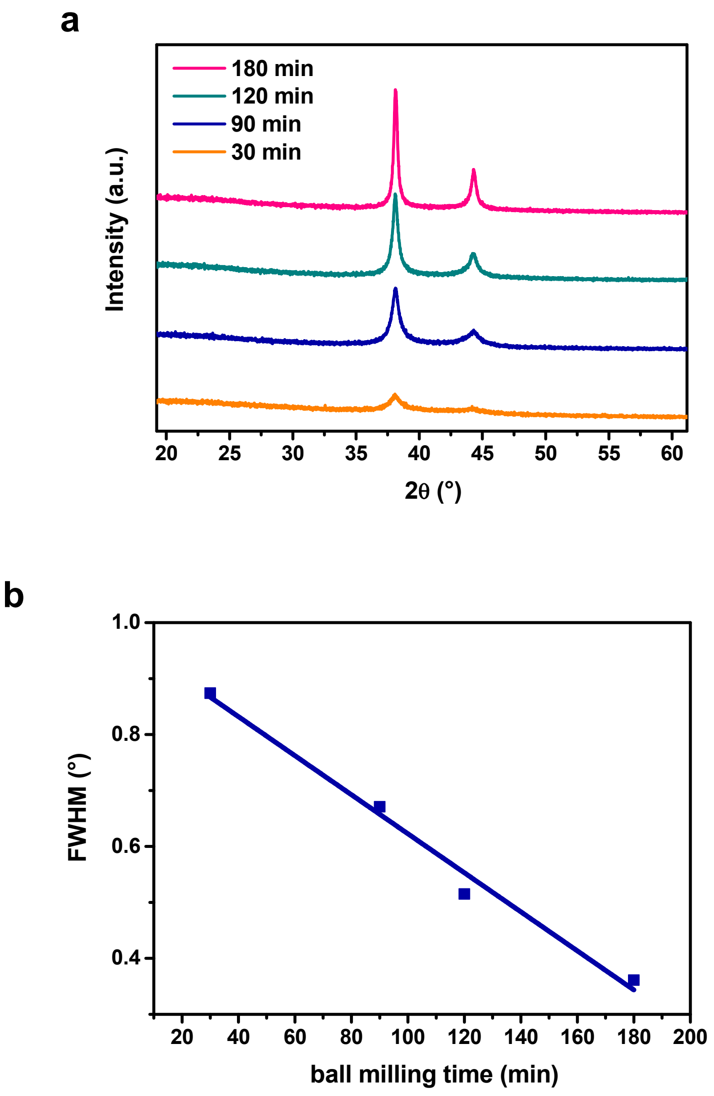

Figure S3. (a) XRD spectra of PNS/$AgNO_3$ samples subjected to varying planetary ball-milling times (increasing from bottom to top). (b) Dependence of the FWHM of the main peak on the milling treatment time.
